# Extraversion and anterior vs. posterior DMN activity during self-referential thoughts

**DOI:** 10.3389/fnhum.2012.00348

**Published:** 2013-01-07

**Authors:** Gennady G. Knyazev

**Affiliations:** Institute of Physiology, Siberian Branch of Russian Academy of Medical SciencesNovosibirsk, Russia

**Keywords:** extraversion, default mode network, EEG, alpha oscillations, independent component analysis

## Abstract

Recent studies show that fronto-posterior electroencephalogram (EEG) spectral power distribution is associated with personality. Specifically, extraversion is associated with an increase of spectral power in posterior cortical regions that overlap with the posterior default mode network (DMN) hub and a decrease of spectral power in anterior regions that overlap with the anterior DMN hub. Although there is evidence that dopaminergic neurotransmission may be involved, psychological processes that underlie these associations remain unclear. I hypothesize that these processes may have something to do with spontaneous self-referential thoughts. Specifically, I hypothesize that in extraverts self-referential thoughts may be associated with an increase of spectral power in the posterior DMN hub, whereas in introverts they may be associated with an increase of spectral power in the anterior DMN hub. After spontaneous EEG registration, participants were asked to fill in a questionnaire describing their thoughts during the registration. An item describing self-referential positive expectations (SRPE) was used to measure individual differences in the intensity of these processes. Source localization and independent component analyses were applied to EEG data to reveal oscillatory activity associated with the anterior and the posterior DMN hubs. Hierarchical regression analysis showed a significant interaction between extraversion scores and anterior vs. posterior DMN alpha activity in predicting individual differences in SRPE scores. In extraverts, high SRPE scores were associated with an increase of alpha power in the posterior DMN hub, whereas in introverts they were associated with an increase of alpha power in the anterior DMN hub. Results are discussed in terms of differential involvement of the two DMN hubs in self-related reward processes in extraverts and introverts.

## Introduction

Extraversion is one of a few major dimensions of personality which consistently appear in most personality models. The view, which currently is most popular, links extraversion with the activity of the brain's dopaminergic (DA) reward system (Depue and Collins, 1999; Smillie et al., 2006). However, investigation of DA neurotransmission in humans requires either invasive measurements or expensive neuroimaging techniques. In this connection, the electroencephalographic (EEG) index of DA neurotransmission, which has been suggested by Wacker et al. (2006), seems very attractive. This suggestion is based on observations of an association between extraversion and posterior vs. frontal EEG activity (Hewig et al., 2004, 2006; Wacker et al., 2006). Wacker et al. (2006) demonstrated that the negative association between extraversion and the frontal minus parietal theta activity, which was observed in the placebo group, was completely reversed in the group that received the selective DA D2 antagonist. Similar effect was observed in alpha band. This finding implies that the fronto- posterior distribution of spectral power may reflect trait-like predispositions which depend on the brain DA functioning. This group of researchers has replicated this finding in several studies [see e.g., meta-analysis by Wacker et al. (2010)]. Moreover, they found an association between the posterior minus frontal slow activity on the one hand and polymorphisms of the DA D2 receptor (Koehler et al., 2011) and enzyme catechol-O-methyltransferase (Wacker and Gatt, 2010) on the other hand. At least one another independent group found a similar association of extraversion with the fronto- posterior spectral power distribution (Knyazev, 2009, 2010; Knyazev et al., 2012a). However, these findings leave unanswered the question about psychological processes that underlie these associations.

It could be noted that the above described associations are in line with some other findings indicating that posterior cortical areas may be more active in extraverts, at least in some circumstances. For example, Yuan et al. (2012) show that posterior cingulate cortices may mediate extraversion-related effect for pleasant stimuli, which essentially results in a decreased threshold for pleasant emotion and an increased threshold for unpleasant emotion. Higher scores on extraversion were found to be associated with higher amplitudes of the P300 component of the ERPs elicited by human faces in parietal cortical regions (Fishman et al., 2011). Phillips et al. (2012) show that monkeys who demonstrate higher levels of exploratory approach behavior have significantly greater gray matter density in the precuneus. On the other hand, there is indirect evidence implying that prefrontal cortical regions might be less active in extraverts. Thus, neural valuation signals in the anterior cingulate cortex and functional coupling of this region with hippocampus and amygdala predict the degree to which future thinking modulates individual preference functions and reduces the rate of delay discounting (Peters and Büchel, 2010); it is known that extraversion is associated with greater discounting (Richards et al., 1999).

### The fronto- posterior spectral power gradient and the default mode network

Because most of the associations between extraversion and posterior vs. frontal EEG were observed in resting conditions (but see Knyazev, 2009), it seems reasonable to suggest that they may relate to psychological processes and respective brain networks which are most active in these conditions. Recent studies have revealed several networks in the brain which are active in the resting state. Most intriguing findings and ideas are associated with the so-called default mode network (DMN). The DMN is a constellation of brain areas which decrease their activity during a wide number of different goal-oriented tasks as compared to passive “rest” tasks (Raichle and Snyder, 2007). Interestingly, several DMN regions are also related to social cognition (Mitchell, 2006; Gobbini et al., 2007). In line with these findings recent studies have revealed DMN abnormalities in autistic patients (Kennedy et al., 2006; Kennedy and Courchesne, 2008) and in patients with social phobia (Gentili et al., 2009). Although EEG has lower spatial resolution than fMRI and has no direct access to deep cortical regions (e.g., DMN's midline cortices), it could be noted that the anterior and the posterior cortical areas, whose oscillatory activity shows correlations with extraversion, overlap with the anterior and the posterior DMN hubs, respectively. The relation of the posterior vs. anterior spectral power distribution to DMN was also hypothesized by Wacker et al. (2010) and Chavanon et al. (2011).

### The anterior and the posterior DMN hubs

The DMN comprises a set of brain regions that are co-activated during passive states and show intrinsic functional correlations with one another. However, there is clear evidence that the brain regions within the DMN contribute specialized functions that are organized into subsystems that converge on hubs (Buckner et al., 2008). Maps of the intrinsic correlations within the default network show that it comprises at least three interacting subsystems. The medial temporal lobe subsystem functions to provide information from prior experiences in the form of memories and associations that are the building blocks of mental simulation; the medial prefrontal cortex (MPFC) subsystem facilitates the flexible use of this information during the construction of self-relevant mental simulations. These two subsystems converge on the precuneus/posterior cingulate cortex (pC/PCC) (Buckner et al., 2008). Partial correlation network analysis suggests that this latter region may play a pivotal role in the DMN (Fransson and Marrelec, 2008). Indeed, PET studies have shown that the metabolic activity is higher in the pC/PCC than all other regions during rest (Gusnard and Raichle, 2001). It could be suggested that being the place of integration of prior experiences with mental simulations, the pC/PCC sustains a sense of self-consciousness that is engaged in self-referential mental thoughts during rest (for reviews see Cavanna and Trimble, 2006; Buckner and Carroll, 2007) and is commonly associated with positive emotions (Koole et al., 2001).

### The anterior DMN hub

It appears that social salience which reflects the relation between others and oneself is processed by the MPFC (Iacoboni et al., 2004; Schmitz et al., 2004; Seger et al., 2004; Han et al., 2005; Mitchell et al., 2005a,b; Ochsner et al., 2005). MPFC is activated during thinking about the complex interactions among people (Buckner et al., 2008). There is ground to suggest that thinking about the complex interactions among people is frequently accompanied by negative emotion. Besides, the anterior mid-cingulate cortex is implicated in the integration of negative affect, pain, and cognitive control (Shackman et al., 2011). In general, being a part of the prefrontal cortex, the MPFC is inevitably involved in conscious planning, decision making, and control functions (see e.g., Luk and Wallis, 2009; Alexander and Brown, 2011). Therefore, these cortices are bound to be reciprocally related to motivational centers, such as amygdala and striatum (Quirk and Beer, 2006; Urry et al., 2006; Goldin et al., 2008; Ochsner and Gross, 2008). Indeed, much evidence shows that, the MPFC controls the accumbens dopamine responses to environmental challenges (e.g., Pascucci et al., 2007) and dopamine release in the MPFC exerts an inhibitory influence on dopamine release in the nucleus accumbens, whereas depletion of mesocortical DA facilitates activation of mesoaccumbens DA release (Deutch et al., 1990; Doherty and Gratton, 1996; King et al., 1997).

### The posterior DMN hub

The pC/PCC cortices, which constitute the posterior DMN hub, are involved in self-centered cognition (e.g., ongoing self-monitoring) and self vs. others discrimination (Vogt et al., 2006). First-person-perspective taking in social interaction and in a language task shows differential activation in the medial aspects of the superior parietal lobe and the right temporo-parietal junction (Vogeley et al., 2001; Vogeley and Fink, 2003). PCC, the retrosplenial, and the medial parietal cortices are implicated in putting self-referential stimuli within a temporal context, linking them to past self-referential stimuli (Northoff et al., 2006). Transcranial magnetic stimulation over the medial parietal region caused a decrease in the efficiency of retrieval of previous judgment of mental self as compared to retrieval of judgment of other, confirming that this region may be a nodal structure in self-representation (Lou et al., 2004). Direct appraisals of self as compared to reflected appraisals recruited PCC (Ochsner et al., 2005). Besides, the right inferior parietal cortex and precuneus may be specifically involved in distinguishing self-produced actions from those generated by others (Ruby and Decety, 2001). It should be borne in mind also that the parietal cortex is activated by emotional stimuli that are not the focus of attention and are therefore perceived mostly unconsciously (Iidaka et al., 2001; Knyazev et al., 2009). Moreover, the parietal cortex is a part of the dorsal (non-conscious) processing stream which contributes to vision-for-action (Milner and Goodale, 1995; Goodale and Milner, 2008) and participates in salience detection (Husain and Nachev, 2007). Non-spatial salience detection functions are particularly associated with the inferior parietal lobe, which in humans consists of novel cortical areas not shared with other primates (Husain and Nachev, 2007). Summing up, existing evidence shows that the anterior DMN hub is involved in mostly conscious modeling, planning, and control functions whereas the posterior hub is involved in mostly unconscious processes that include self-representation, emotion, and salience detection. In the context of the present study, it is interesting to note that according to Gray's (1999) theory, salience detection is the main function of the dopaminergic reward system.

### The anterior and posterior DMN hubs and DA

Because the association between extraversion and posterior vs. anterior EEG activity is mediated by dopamine, it is interesting to note that the anterior and the posterior DMN hubs appear to be differently susceptible to dopaminergic influences. Generally, dopaminergic effects appear to be more pronounced in the posterior than in the anterior hub or these effects could be of opposite directions. Many of these observations have been made on Parkinson's disease (PD) patients. Thus, van Eimeren et al. (2009) show that patients with mild to moderate PD (not taking medication) and healthy controls showed comparable deactivation of the MPFC, but different deactivation in the pC/PCC. Compared with controls, PD patients not only showed less deactivation of the pC/PCC, they even demonstrated a reversed pattern of activation and deactivation. Dopamine medication appears to restore the normal pattern of task-related deactivation in the posterior DMN hub. Thus, PD patients taking placebo only deactivated the ventral MPFC during a facial emotion recognition task. They failed to deactivate the posterior midline and lateral parts of DMN. After levodopa administration, this network was restored conjointly with the improvement of motor dysfunction in PD patients (Delaveau et al., 2010). In another study, PD patients were scanned twice, once while on their DA medication (ON condition) and once after medication withdrawal (OFF condition). Higher activation in the precuneus was found in the ON condition (Dusek et al., 2012). Krajcovicova et al. (2012) using the daily levodopa equivalent dose in cognitively unimpaired PD patients as a covariate observed an enhanced functional connectivity of the DMN in the posterior cingulate cortex during a cognitive task. Similar effects were observed not only in PD patients, but also in healthy older adults who compared with younger adults showed diminished fMRI deactivations in pC/PCC during memory recognition. In younger adults, greater task-induced deactivation in this region was associated with higher dopamine synthesis capacity (as measured by the radiotracer 6-[18F]-fluoro-L-*m*-tyrosine). The authors suggest that DA system helps modulate the posterior DMN hub activity in younger adults and that alteration to the DA system may contribute to age-related changes in working memory function (Braskie et al., 2011). Healthy adult subjects that received methylphenidate (a stimulant drug that amplifies dopaminergic signaling in the brain) had increased deactivation during working memory and visual attention tasks in the insula and the PCC (but not in the MPFC) than the group of subjects who received placebo (Tomasi et al., 2011).

Some authors observed opposite dopamine-related effects in the posterior and the anterior DMN hubs. Thus, Tomasi et al. (2009) assessed the relationship between DA transporters (DAT, which regulate extracellular dopamine in the brain) in striatum (measured with positron emission tomography and [11C] cocaine used as DAT radiotracer) and brain activation and deactivation during a parametric visual attention task (measured with BOLD fMRI) in healthy controls. DAT availability in caudate and putamen had a negative correlation with deactivation in ventral parietal regions of the DMN (precuneus, BA7) and a positive correlation with deactivation in the ventral anterior cingulate gyrus (BA24/32). Similarly, Asanuma et al. (2006) show that, levodopa therapy was associated with significant metabolic increases in the precuneus (BA7) but decreases in the MPFC. This evidence appears to suggest that dopamine may exert inhibitory effect on the anterior and excitatory effect on the posterior DMN hub. The former effect is in line with animal data showing that DA increases the threshold for spike firing and exerts an inhibitory action in the prefrontal cortex (Geijo-Barrientos and Pastore, 1995).

In sum, the evidence presented in the previous sections appears to suggest that although both the anterior and the posterior DMN hubs are involved in self-centered and social cognition and are co-activated during passive states, they are associated with rather different functions. The anterior DMN is more involved in integration, planning, and control functions, which are mostly conscious and are reciprocally related to dopaminergic reward processes. The posterior DMN is more involved in self-representation and salience detection. The latter processes are mostly unconscious and are positively related to dopaminergic reward processes. The former processes are less and the latter processes are more pronounced in extraverts than in introverts.

### The present study

I hypothesize that the association between extraversion and the resting state posterior vs. frontal EEG activity is mediated by DMN-related spontaneous self-referential processes in such a way that in more extraverted individuals these processes are associated with an increase of spectral power in the posterior DMN hub, whereas in more introverted individuals they could be associated with an increase of spectral power in the anterior DMN hub. In this study, I aimed to obtain EEG records during unconstrained mind-wandering and to test whether extraversion moderates the associations between the prevalence of relevant self-referential thoughts and EEG spectral power within the anterior and the posterior DMN hubs. The existence of an association between self-referential thoughts and EEG spectral power within the DMN has been shown previously (Knyazev et al., 2011, 2012b). The choice of a relevant measure of extraversion and a relevant measure of self-referential thoughts was guided by the hypothesis linking these processes with dopaminergic transmission and social cognition. Depue and Collins (1999) argue that extraversion can be subdivided into two subfactors: affiliation and agency. They propose a dopaminergic basis for the agency facet of extraversion (i.e., a motivational disposition that comprises social dominance, enthusiasm, energy, assertiveness, ambitiousness, and achievement striving). Keeping in mind that the DMN, which is the main focus of this study, is supposedly involved in self-referential processes in the context of interpersonal relationships (e.g., Mitchell, 2006), assertiveness appears to be the facet of extraversion which best captures both the agentic properties of this dimension and its projection onto the space of interpersonal relationships. High assertiveness scorers are independent, dominant, and stand up for their rights. They tend to be at the center of attention at meetings. Low scorers are humble, timid, submissive, and disinclined to take initiative in interpersonal situations, and may be easily imposed upon (Eysenck and Wilson, 2000). With regard to the relevant kind of self-referential thoughts, I intended to capture an aspect of anticipation of a positive reinforcement that is peculiar to extraverts and is supposedly mediated by the dopaminergic reward system.

## Methods

### Subjects

Resting EEG data were collected in 60 healthy volunteers (32 men and 28 women; age range 17–30 years, mean = 20.4, *SD* = 2.5), mostly university students. All applicable subject protection guidelines and regulations were followed in the conduct of the research in accordance with the Declaration of Helsinki. All participants gave informed consent to the study. The study has been approved by the Institute of Physiology ethical committee.

### Instruments and procedures

Participants were seated in a soundproof dimly illuminated room and did not receive any instruction. The spontaneous EEG registration lasted about 6 min and included alternating 2 min intervals with eyes open and eyes closed. Only the eyes closed condition was used in this study because previous research has shown that self-referential thoughts correlate with EEG spectral power in the eyes closed, but not in the eyes open condition (Knyazev et al., 2011). Just after the EEG registration participants were asked to fill in a brief (35 items) spontaneous thoughts questionnaire (STQ) which described different aspects of their state, thoughts, and feelings during the registration. All items were measured on a five-point Likert scale. Factor analysis of all questionnaire items (principal components factor analysis with varimax rotation) showed that a four-factor solution best fitted the data. Accordingly, four scales were created that described nervousness/negative emotion/lack of positive emotion (NE, example items: “felt nervous,” “experienced negative emotions,” “was calm and relaxed”—reverse scoring, “liked the procedure”—reverse scoring, Cronbach's alpha = 0.84); self-referential thought (SRT, example items: “thought about something pleasant that is going to happen to me in the near future,” “recollected episodes from my own life,” “most of the time, thoughts of my recent past recurred to me,” “most of the time, I was absorbed in my private thoughts,” Cronbach's alpha = 0.69); arousal level (example items: “was almost asleep”—reverse scoring, “was quiet and relaxed”—reverse scoring, “was somewhat heated,” “was very excited,” Cronbach's alpha = 0.72); attention to environment (ATT, “my attention was mostly directed to external stimuli,” “most of the time, I listened to sounds and skin sensations,” “did not pay any attention to external stimuli”—reverse scoring, Cronbach's alpha = 0.65). The SRT scale (SRTS) was used to measure individual differences in mental processes that are presumably related to DMN activity. Besides, for the purpose of this study, I additionally used the first item from this scale (see above), which describes self-referential positive expectations (hereafter SRPE). After filling in the questionnaire subjects participated in experiments which are not described here. After the experiments they filled in a set of personality questionnaires and were debriefed. Facets of Extraversion were measured by respective scales from the Eysenck Personality Profiler (EPP, Eysenck and Wilson, 2000; Knyazev et al., 2004). Assertiveness scale (Cronbach's alpha = 0.78) consisted of 20 items. Example item: “Do you find it difficult to get rid of a salesperson who is persistent and wasting your time?”. Activity (Cronbach's alpha = 0.73) and Sociability (Cronbach's alpha = 0.81) scales were additionally used in order to test the specificity of observed effects.

### EEG recording

EEG data were recorded using 32 silver-silver chloride electrodes mounted in an elastic cap on the positions of the international 10–20 system. The signals were amplified with a multichannel biosignal amplifier with a gain of 250 and a bandpass 0.05–70 Hz, −6 dB/octave and continuously digitized at 300 Hz. All recordings were performed using a fronto-central electrode as ground and electronically linked mastoid electrodes as reference. The horizontal and vertical electrooculogram was registered simultaneously. Electrode impedances were at or below 5 kΩ for all electrodes used in the analysis. EEG data were artifact-corrected using ICA via EEGLAB toolbox (http://www.sccn.ucsd.edu/eeglab/) retaining minimally 20 out of 30 components.

### 3D source reconstruction

To determine the cortical sources of EEG activity, sLORETA (Pascual-Marqui, 2002) was applied to the data. sLORETA uses a three-shell spherical head model registered to the digitized Talairach and Tournoux (1988) atlas. The solution space is restricted to cortical gray matter and parahippocampal areas. sLORETA yields images of standardized current source density of a total of 6430 voxels at 5-mm spatial resolution. Artifact-free epochs of 1.7 s duration were supplied for cross-spectrum calculation in sLORETA. The number of epochs varied in different subjects from 85 to 210. Subsequently current source densities of delta (2–4 Hz), theta (4–8 Hz), alpha (8–12 Hz), beta (12–30 Hz), and gamma (30–45 Hz) oscillations were estimated in sLORETA. The regularization factor was set at 1/100 (Congedo, 2006).

### Independent component analysis

In this study, I used the group spatial ICA, because it directly estimates components that are consistently expressed in the population, involves the least amount of user interaction and is straightforward to compare with the existing framework for group ICA of fMRI data (Calhoun et al., 2001). First, current source density estimates for the five EEG frequency bands were calculated using sLORETA. For each frequency band separately, each trial's sLORETA images were converted into the neuroimaging informatics technology initiative (NIFTI) format using modified by the first author LOR2SPM function by Pakhomov (http://www.ihb.spb.ru/~pet_lab/L2S/L2SMain.htm). Next, group spatial ICA was applied to sLORETA images in a fashion that is routinely used in fMRI research. Spatial ICA was performed using the Group ICA for fMRI Toolbox (GIFT, Version 1.3i; http://icatb.sourceforge.net/), using methods and algorithms described elsewhere (Calhoun et al., 2001, 2004). Briefly, a single ICA was performed at the group level after subject-wise data concatenation. Obtained independent components (ICs) were back reconstructed to produce single-subject time courses and spatial maps from the raw data matrix (Calhoun et al., 2001). The minimum description lengths (MDL) criterion was used to estimate the number of extracted components from the data. Basing on these estimates, 20 components were extracted. One-sample T-tests in SPM8 were used to assess the statistical significance of each component. Each subject's respective component image (*z*-score spatial map) was entered into a second-level random-effects analysis and assessed statistically using a threshold of P_FDR_ = 0.05 (whole-brain corrected) and minimum cluster size of 8 contiguous voxels. Only the components that were statistically significant across subjects were used in further analyses.

For each respective set of ICA results, ICs were spatially correlated with an anatomically defined template and were ranked according to a “highest correlation” criterion with this anatomy. I created two templates using the Wake Forest Pick atlas toolbox (http://www.fmri.wfubmc.edu/) (Maldjian et al., 2004). The anterior DMN (ADMN) template included the medial frontal and the superior frontal gyrus (BAs 8/9/10) and the anterior cingulate cortex (BAs 11/24/32). The posterior DMN (PDMN) template included the posterior parietal cortex (BA 7), the occipitoparietal junction (BA 39), the posterior cingulate, and the precuneus. For the analysis of associations between SRPE scores and inter-individual variation in components' intensity, for each of 20 ICs generated for each frequency band, all positive voxel values in a respective (*z*-score-transformed) independent component image were summed for each subject. These values were further used as is described below. This approach to capturing inter-individual differences is based on the following. After initial decomposition on all concatenated datasets at once, the components are back reconstructed in each individual subject. After that, each component is more pronounced in some subjects and may be weak or absent in others. If a component is strongly pronounced, respective brain areas will have high positive *z*-score values. Therefore, summing and comparing across subjects the positive values in *z*-transformed spatial maps allows revealing individual differences in intensity of this component. This method is described in the paper by Allen et al. (2011). For more details see Knyazev et al. (2011, 2012a,b).

### Statistical analysis

For the analysis of moderation effects a combined measure of PDMN vs. ADMN activity (hereafter P/ADMN) was created. The PDMN_IC_ (hereafter subscript IC denotes the independent component that showed the highest spatial correlation with a respective template) and ADMN_IC_ scores were converted to *z*-scores and ADMN_IC_ scores were subtracted from PDMN_IC_ scores. The resulting measure represented dimension running from low PDMN/high ADMN activity to high PDMN/low ADMN activity.

To test for moderation, regression analyses were specified for the combination of moderator (i.e., extraversion) and factor (i.e., P/ADMN) as predictors of SRTS or SRPE scores. Following guidelines on testing moderator models outlined by Baron and Kenny (1986), predictor variables were entered hierarchically in the following order: (1) main effects for factor tested (i.e., P/ADMN) and proposed moderator variable (i.e., extraversion); (2) the two-way interaction between the factor and the moderator. To test interactions (or moderation effects) involving continuous variables, I converted all continuous variables to *z*-scores, following the suggestion by Aiken and West (1991). To gain an understanding of the overall pattern of the interaction, regression slopes were plotted graphically at high (0.5 SD) and low (−0.5 SD) values of the moderator.

## Results

Comparison of EPP scales' means of the current sample with a normative Russian sample (*N* = 576, Knyazev et al., 2004) showed no significant differences on any of the nine personality characteristics. Sociability and Activity correlated positively with SRTS (*r* = 0.32, *p* = 0.017 and *r* = 0.39, *p* = 0.002, respectively) and SRPE (*r* = 0.34, *p* = 0.009 and *r* = 0.38, *p* = 0.003, respectively) and negatively with ATT (*r* = −0.28, *p* = 0.038 and *r* = −0.27, *p* = 0.039, respectively). Assertiveness did not show significant correlations with STQ scales.

There was a significant interaction of Assertiveness with P/ADMN in prediction of SRTS scores, *B* = −0.31, *T*_(58)_ = −2.14, *p* = 0.037. There were no significant moderation effects of Assertiveness in other frequency bands. Moderation effects for Activity and Sociability were not significant for all frequency bands.

**Figure 1:**
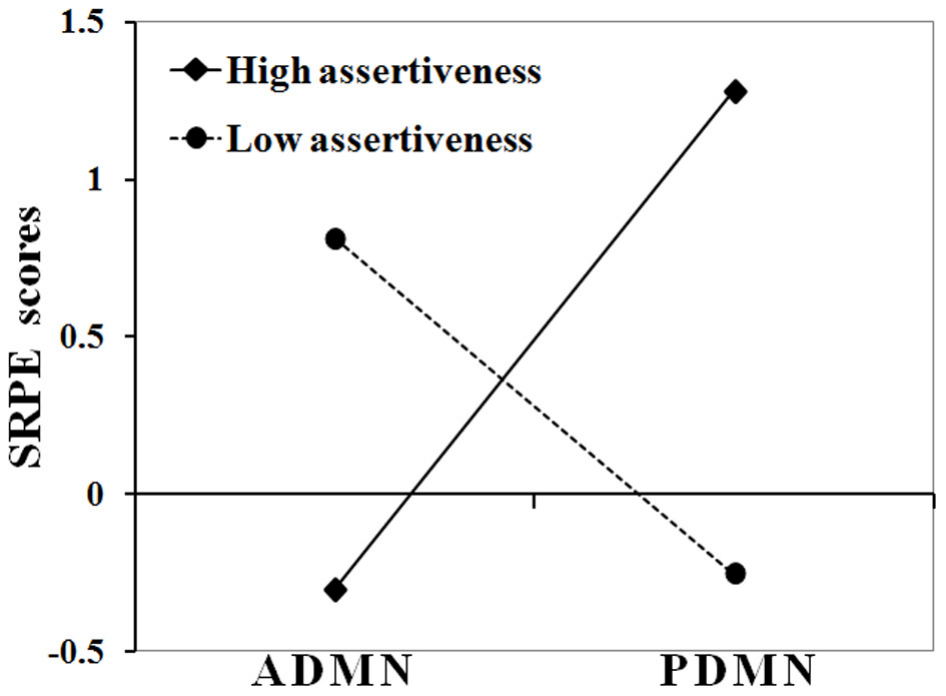
**Interaction between Assertiveness and P/ADMN alpha activity in their effect on SRPE scores.** Ordinate axis represents SRPE *z*-scores; abscissa axis runs from low PDMN and high ADMN alpha activity to high PDMN and low ADMN alpha activity; solid line represents the group of subjects with high assertiveness (>0.5 SD); dashed line represents the group of subjects with low assertiveness (<−0.5 SD).

Next, moderation analyses were conducted for all SRTS items separately. Only for the first item (i.e., SRPE) its relationship with alpha band P/ADMN activity was significantly moderated by Assertiveness, *B* = −0.71, *T*_(58)_ = −3.52, *p* = 0.001. Figure 1 shows regression slopes of alpha band P/ADMN on SRPE scores in subjects with high (+0.5 SD, *N* = 17) and low (−0.5 SD, *N* = 15) Assertiveness.

**Figure 2:**
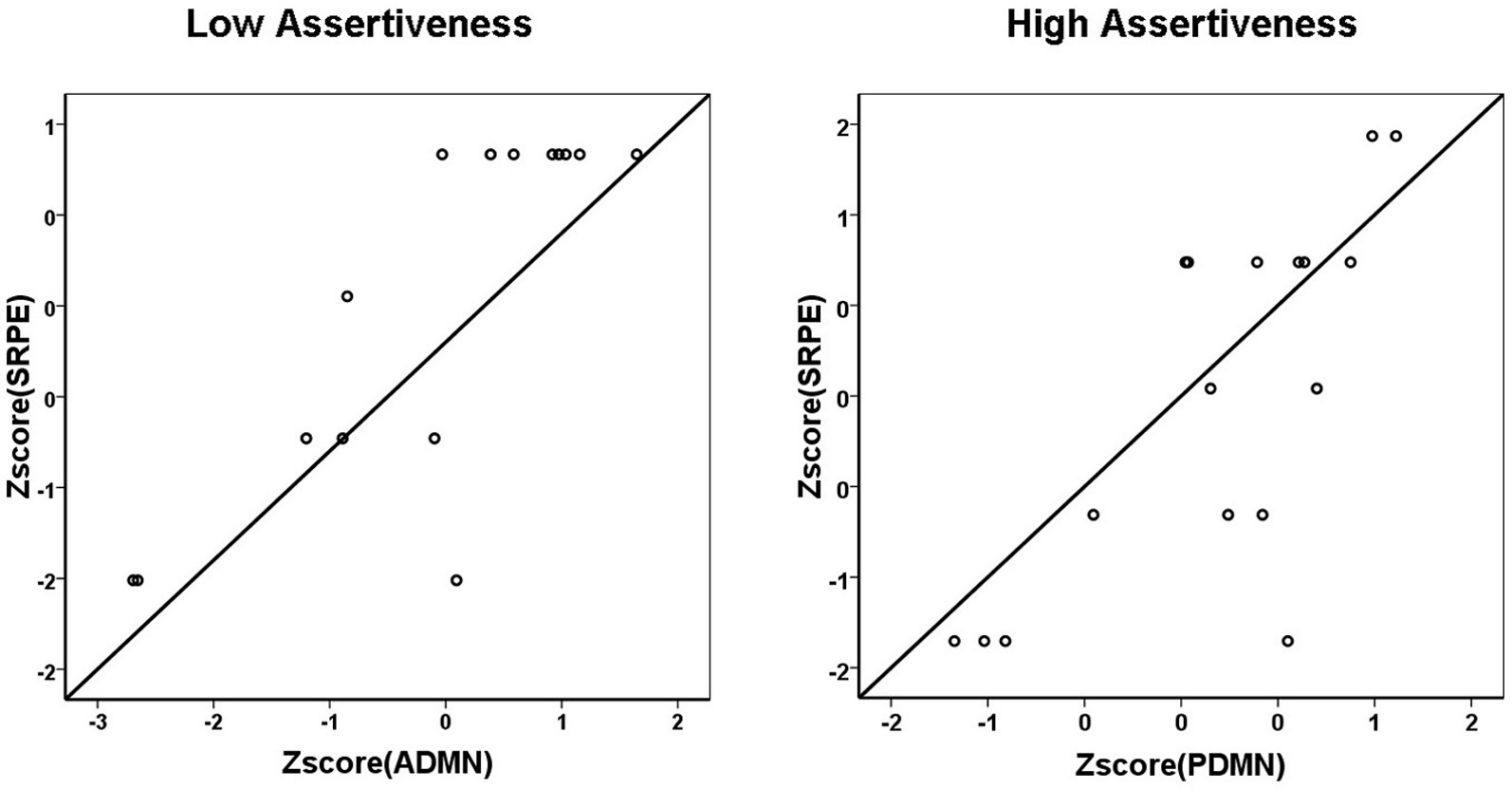
**Scatter-plots of the relationships between SRPE scores and alpha activity in the ADMN in low (left panel) and in the PDMN in high (right panel) Assertiveness scorers**.

As this figure shows, in high Assertiveness scorers, high SRPE scores were associated with a prevalence of alpha activity in the posterior DMN hub, whereas in low Assertiveness scorers they were associated with a prevalence of alpha activity in the anterior DMN hub. *Post hoc* analyses showed that in high Assertiveness scorers (+0.5 SD, *N* = 17), SRPE scores significantly correlated with PDMN (*r* = 0.74, *p* = 0.001), but not with ADMN (*r* = 0.26, *p* = 0.313) alpha activity, whereas in low Assertiveness scorers (−0.5 SD, *N* = 15), SRPE scores significantly correlated with ADMN (*r* = 0.81, *p* < 0.001), but not with PDMN (*r* = −0.01, *p* = 0.969) alpha activity. Figure 2 shows scatter-plots of the relationships between SRPE scores and alpha activity in the ADMN in low and in the PDMN in high Assertiveness scorers.

**Figure 3:**
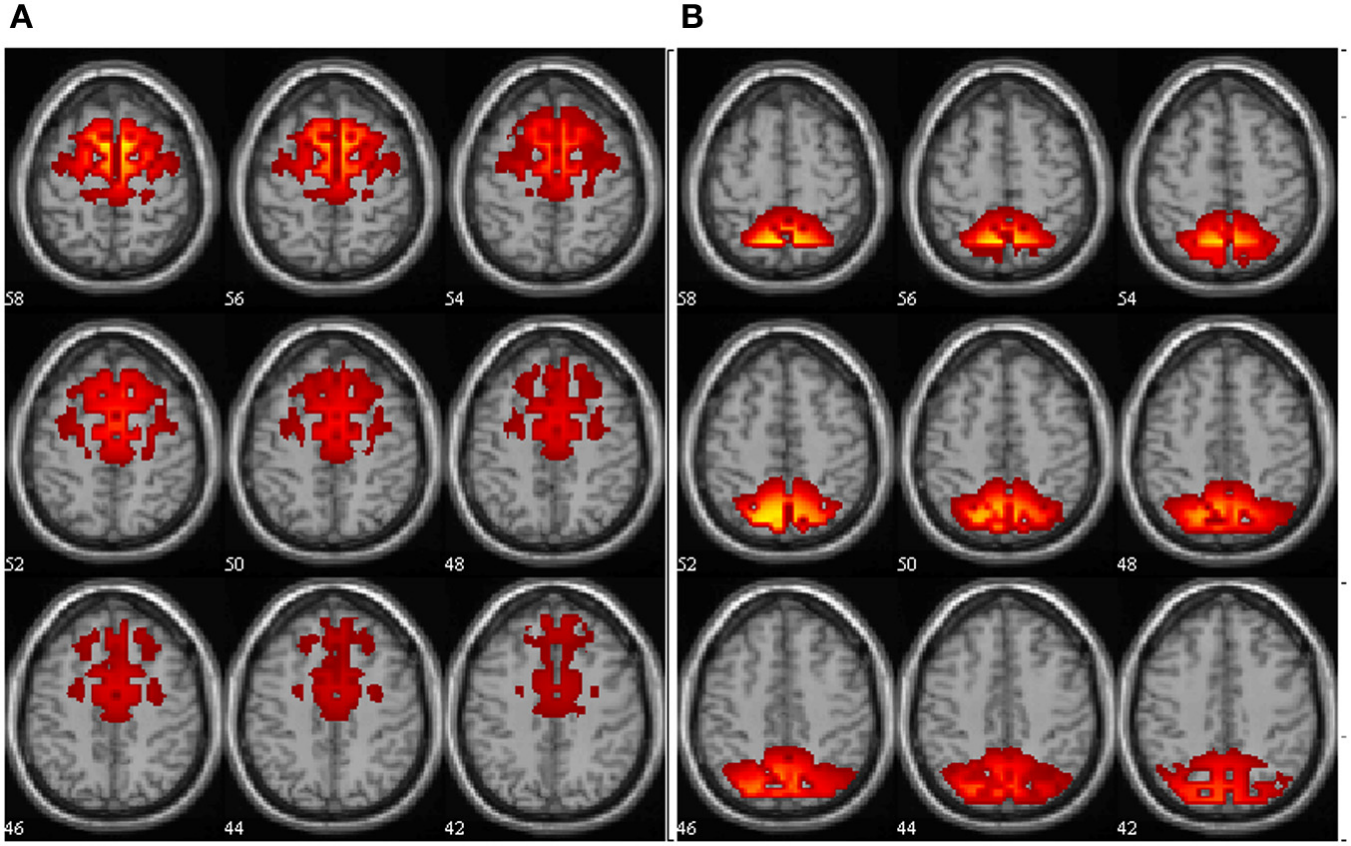
**Anatomy of the ADMN (A) and PDMN (B) alpha band components.** Spatial maps are scaled in *z*-scores. The slices are presented at axial anatomical plane with numbers representing the slice position in mm relative to zero-point.

Figure 3 shows anatomy of the ADMN and PDMN alpha components.

## Discussion

In line with our hypothesis, in more extraverted individuals, spontaneous self-referential thoughts were associated with an increase of spectral power in the posterior DMN hub, whereas in more introverted individuals they were associated with an increase of spectral power in the anterior DMN hub. In our study, these effects were observed in the alpha band of frequencies. It should be noted that in most recent studies, most prominent extraversion-related differences in cortical distribution of spectral power were noted in low frequencies (predominantly theta) (Wacker et al., 2006, 2010; Wacker and Gatt, 2010; Knyazev et al., 2012a). However, differences in the alpha band of frequencies have also been repeatedly described (Hewig et al., 2004, 2006; Wacker et al., 2006; Knyazev, 2009, 2010). Moreover, substantial evidence has been accumulated indicating that DMN-related processes may be specifically positively associated with alpha and beta oscillations. Thus, Jann et al. (2010) note that BOLD correlates of electrical activity in the alpha and beta frequency bands display striking similarity with the DMN. Mantini et al. (2007) showed that DMN and dorsal attentional network have strong relationship with alpha and beta rhythms, albeit in opposite directions, with the former showing positive and the latter showing negative correlations. Jann et al. (2009) show that the BOLD correlates of global EEG synchronization in the alpha frequency band are located in brain areas involved in the DMN. Jann et al. (2010) report DMN activity to be associated with increased alpha and beta1 band power. Sadaghiani et al. (2010) showed that global field power of upper alpha band oscillations is positively correlated with activity in a network overlapping with the DMN and is negatively correlated with activity in the dorsal attention network. Brookes et al. (2011) identified the DMN using magnetoencephalographic data filtered into the alpha band. Knyazev et al. (2011, 2012b) found that alpha band spatial patterns simultaneously showed a considerable overlap with the DMN and high correlations with presumptive DMN-function-related outcomes. In sum, this evidence suggests that alpha oscillations appear to be positively related to DMN and negatively to attentional networks. Contrary to that, theta oscillations show negative correlations with the DMN (Scheeringa et al., 2008). It could be suggested that extraversion-related differences in cortical distribution of spectral power could be observed in different frequency bands (see e.g., Knyazev, 2009); and different frequency bands contribute to different aspects of extraversion-cortical activity associations. Spontaneous self-referential processes appear to be most prominently related to alpha activity and, hence, extraversion-related differences in cortical distribution are observed in this very band.

As has been described in the section “Introduction,” the anterior and the posterior DMN hubs may have different contributions to DMN-related functional outcomes. Specifically, the anterior hub is more involved in modeling the complex social relations. These processes are frequently accompanied by negative emotion and appear to be reciprocally related to nucleus accumbens DA neurotransmission. The posterior hub is more involved in self-centered cognition and salience detection. These processes are commonly associated with positive emotions (Koole et al., 2001) and appear to be positively related to dopaminergic neurotransmission. It appears that spontaneous self-referential thoughts, particularly in the context of anticipation of a positive reinforcement, predominantly engage the posterior DMN hub in extraverts, but the anterior DMN hub in introverts. The two other facets of extraversion (i.e., sociability and activity) did not show significant moderation effects. This could be explained by the fact that sociability is not probably associated with DA neurotransmission (Depue and Collins, 1999), whereas activity is not specifically related to social cognition, which is presumably the main focus of DMN.

Interestingly, a recent study showed that in representatives of a more Western culture (Russia), spontaneous self-referential processes were accompanied by enhanced alpha oscillations in the posterior DMN hub, whereas in representatives of a more Eastern culture (Taiwan) they were accompanied by enhanced alpha oscillations in the anterior DMN hub (Knyazev et al., 2012b). Cross-cultural studies show that Eastern populations are generally lower on extraversion than Western populations (see e.g., Allik and McCrae, 2004), but it has to be revealed in the future whether personality or other cross-cultural variables, such as individualism/collectivism underlie the observed cross-cultural differences.

Some limitations of this study need to be discussed. One methodological limitation is that EEG source localization and ICA were performed on the basis of a somewhat sparse 32 electrodes array. Numerous studies show that localization accuracy improves with increasing the number of recording electrodes (Krings et al., 1999; Laarne et al., 2000; Lantz et al., 2003). ICA decomposition methods generally also require sufficient number of electrodes for reliable and valid component extraction. 32 electrodes may be sufficient, however, so long as there is approximately homogenous scalp coverage (Lantz et al., 2003; Congedo, 2006), as is the case in this study. A simulation study has shown that 32 electrodes array in combination with the method that was used in the current study are sufficient for accurate localization of cortical sources and revealing their time dynamics, frequency characteristics, and between-subject variability (Knyazev et al., 2012a). Moreover, in a recent study (Knyazev et al., 2012b), we replicated the Knyazev et al.'s (2011) findings using denser (64 and 132) electrode arrays.

Another concern relates to the fact that sLORETA produces smooth solutions resulting in many correlated voxels which then are submitted for spatial ICA. The correlated voxels will be combined into one extended component with low spatial resolution. This could be a serious limitation when two closely spaced processes are to be distinguished (see e.g., simulation in Knyazev et al., 2012a). However, this limitation is not important in this study because the anterior and the posterior DMN hubs are situated far apart from each other.

Thus, it could be summarized that in extraverted individuals, spontaneous self-referential thoughts are accompanied by enhanced alpha activity within the posterior DMN hub, whereas in introverted individuals they are accompanied by enhanced alpha activity in the anterior DMN hub. There is a solid ground to suggest that these effects are mediated by dopaminergic neurotransmission, because a number of studies by Wacker and colleagues have shown that extraversion-related posterior vs. anterior EEG asymmetries are associated with the dopaminergic system (Wacker et al., 2006; Wacker and Gatt, 2010; Koehler et al., 2011).

### Conflict of interest statement

The author declares that the research was conducted in the absence of any commercial or financial relationships that could be construed as a potential conflict of interest.
